# Spatiotemporal Evolution of *Calophaca* (Fabaceae) Reveals Multiple Dispersals in Central Asian Mountains

**DOI:** 10.1371/journal.pone.0123228

**Published:** 2015-04-07

**Authors:** Ming-Li Zhang, Zhi-Bin Wen, Peter W. Fritsch, Stewart C. Sanderson

**Affiliations:** 1 Key Laboratory of Biogeography and Bioresource in Arid Land, Xinjiang Institute of Ecology and Geography, Chinese Academy of Sciences, Urumqi, China; 2 Institute of Botany, Chinese Academy of Sciences, Beijing, China; 3 Department of Botany, California Academy of Sciences, Golden Gate Park, San Francisco, California, United States of America; 4 Shrub Sciences Laboratory, Intermountain Research Station, Forest Service, U.S. Department of Agriculture, Provo, Utah, United States of America; Institute of Botany, CHINA

## Abstract

**Background:**

The Central Asian flora plays a significant role in Eurasia and the Northern Hemisphere. *Calophaca*, a member of this flora, includes eight currently recognized species, and is centered in Central Asia, with some taxa extending into adjacent areas. A phylogenetic analysis of the genus utilizing nuclear ribosomal ITS and plastid *trnS-trnG* and *rbcL* sequences was carried out in order to confirm its taxonomic status and reconstruct its evolutionary history.

**Methodology/Principal Finding:**

We employed BEAST Bayesian inference for dating, and S-DIVA and BBM for ancestral area reconstruction, to study its spatiotemporal evolution. Our results show that *Calophaca*is monophyletic and nested within *Caragana*. The divergence time of *Calophaca* is estimated at ca. 8.0 Ma, most likely driven by global cooling and aridification, influenced by rapid uplift of the Qinghai Tibet Plateau margins.

**Conclusions/Significance:**

According to ancestral area reconstructions, the genus most likely originated in the Pamir Mountains, a global biodiversity hotspot and hypothesized Tertiary refugium of many Central Asian plant lineages. Dispersals from this location are inferred to the western Tianshan Mountains, then northward to the Tarbagatai Range, eastward to East Asia, and westward to the Caucasus, Russia, and Europe. The spatiotemporal evolution of *Calophaca* provides a case contributing to an understanding of the flora and biodiversity of the Central Asian mountains and adjacent regions.

## Introduction

The Himalaya Range and Qinghai Tibet Plateau (QTP), as well as the associated Pamir and Tianshan mountains in Asia, have developed unparalleled alpine and montane floras, and therefore often have served as a source for plants adapted to higher elevations, even affecting the Alps of Europe[1–6]. Arid portions of these mountain ranges have developed drought adapted montane floras and enabled xeric plants to disperse more widely over geologic time [7,8]. However, this issue has received surprisingly little examination by modern methods of phylogenetic and biogeographical reconstruction [4].

From geological history perspectives, at the uplift of the QTP and withdrawal of the inland Paratethys Sea during the late Eocene [9], pronounced aridity appears to have begun in Central Asia [9], followed by a switch to monsoonal circulatory patterns, resulting in arid winter monsoon winds in the lee of the QTP in Central Asia by late Oligocene or early Miocene [10,11]. These circumstances resulted in the evolution of a well developed dryland Central Asian flora, which has yielded different evolutionary hypotheses, such as origins locally or from the Mediterranean, Africa, East Asia, or the QTP [1,4,6,7,12]. It appears that some of the elements of this flora proved to be preadapted as aridity increased across Eurasia in connection with global cooling during the Miocene and Pliocene [11,13]. Case studies of members of the Central Asian flora to test these evolutionary hypotheses, such as that of *Artemisia* (Asteraceae) [13], with linkage to paleogeography and paleoclimate events, and providing exact descriptions of spatiotemporal evolution, have to date been very few.

**Fig 1:**
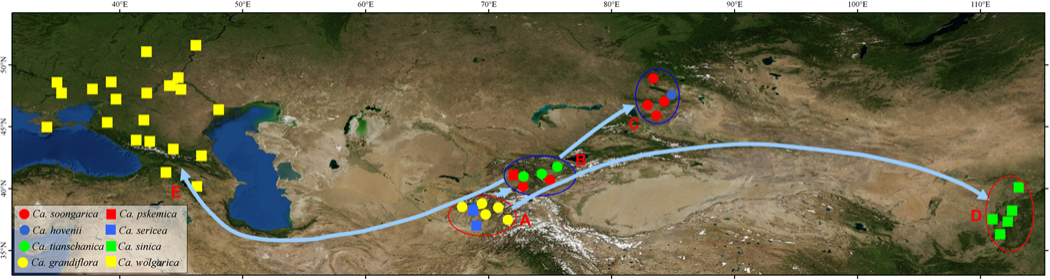
Distribution of *Calophaca* species as estimated from our field investigations in China, and floras and herbaria (PE, XIJI, LE, MW, MAH). The place of origin of the genus, the Pamir—western Tianshan, and dispersal routes in three different directions are illustrated.

*Calophaca* Fisch. ex DC. is an ideal taxonomic group to investigate the Central Asian flora. It includes small trees or shrubs and eight currently described species distributed in semiarid montane and steppe environments, chiefly in Central Asia, with one species extending westward to southeastern Europe and Russia, and another in northern China [14–18]. The distribution of *Calophaca* occupies several global biodiversity conservation hotspots [19], i.e., the Pamir and western Tianshan mountains, the Caucasus, and East Asia, see Fig 1.

Regarding *Calophaca* systematics, Borissova [14] classified *Calophaca* in two series, *Pubiflorae* Boriss., with pubescence on the outside of the standard, and *Glabriflorae* Boriss., with the standard glabrous. *Calophaca* ser. *Glabriflorae* contains two species, *C*. *grandiflora* and *C*. *sericea*, and *C*. ser. *Pubiflorae* contains all of the remaining species of the genus except for *C*. *sinica*. Gorbunova [15] elevated these series as sections: sect. *Glabriflora* and sect. *Calophaca* (Table 1). According to traditional taxonomy [20], the genera *Calophaca*, *Halimodendron*, and *Caragana* belonged to tribe Galegeae. However, these genera were recently transferred to tribe Hedysareae on the basis of molecular evidence [21]. Chromosome data shows that *Calophaca* possesses a chromosome base number of *x* = 8, similar to *Caragana* and *Astragalus*. Of the species of the genus that have been sampled for chromosome number, *C*. *wolgarica*, *C*. *kultiassovii* (= *C*. *tianschanica*), and *C*. *sinica* are diploid [22–24], and *C*. *soongorica* is tetraploid [24]. Li et al. [24] and Chang et al. [25] studied the chromosomes, leaf epidermal features, pollen, carpodermis, and distribution of *C*. *sinica* and *C*. *soongorica*. They hypothesized that the narrow present distribution of *C*. *sinica* resulted from a gradual contraction of its distribution range accompanying the intensification of aridity since the Tertiary [25]. Considering the higher species density of *Calophaca* in Central Asia, some authors have proposed that this genus is a Tertiary relict from the region [25,26]. Molecular approaches coupled with phylogenetic and biogeographical reconstruction may shed more light on the still obscure systematic and evolutionary issues in *Calophaca* and Central Asian biodiversity, however, these have been lacking so far.

**Table 1:** Voucher information for *Calophaca* and four genera as the outgroups.

Taxon	Voucher	Source	GenBank accession number (ITS, *rbcL*, *trnS-G*)
***Calophaca***			
**Sect. *Glabriflora* (Boriss.) Gorb.**			
*Calophaca grandiflora* Regel	M.G. Vasileva 5.8.1983 (MW)	Between rivers Kyzylsu and Yakhsu, Kushtek, S. Tajikistan	KP862569, KP862557, KP862545
*C*. *sericea* B. Fedtsch. ex. Boriss.	E.A. Ra 28.7.1949 (LE)	Pamir, Tajikistan	KP862571, KP862559, KP862547
**Sect. *Calophaca***			
*C*. *tianshanica* (B. Fedtsch.) Boriss.	I.I. Rusanovich 6.06.1989 (MHA)	Algabas, Shymkent, Kazakhstan	KP862578, KP862566, KP862554
*C*. *pskemica* Gorbunova	L. Popova 7.6.1977 (MW)	Sandalash, Pskem, Kyrgistan	KP862581,————,————
*C*. *soongorica* Kar. & Kir.	E.E. Pyoahobeq & L.A. Kpamapehko 5-14-1984 (PE)	Semiipalatinskaya, Kazakhstan	FJ537288, FJ537236, FJ537189
*C*. *soongorica* Kar. & Kir.1	H.X. Zhang et al.20110502 (XIJI)	Tacheng, Xinjiang, China	KP862574, KP862562, KP862550
*C*. *soongorica* Kar. & Kir. 2	H.X. Zhang et al. 20110602 (XIJI)	Tacheng, Xinjiang, China	KP862575, KP862563, KP862551
*C*. *soongorica* Kar. & Kir. 3	M. Pimenova, L. Kamenskih, L. Sdobnina 21.7.1975 (MW)	W. Tarbagatay, Saysu, Kazakhstan	KP862576, KP862564, KP862552
*C*. *soongorica* Kar. & Kir. 4	V.I.Grubov 25.06.1986 (LE)	Ili-Balkhash, Ayagoz, Kazakhstan	KP862577, KP862565, KP862553
*C*. *hovenii* Schrenk.	E. Kluykov 22.8.1979 (MW)	Tarbagatay, Urzharsky, Samipalatinsk, Kazakhstan	KP862570, KP862558, KP862546
*C*. *wolgarica* (L.f.) DC. 1	M.G. Pimenov 10.06.2005 (MW)	Ergeney, Kalmykia, Russia	KP862579, KP862567, KP862555
*C*. *wolgarica* (L.f.) DC. 2	N. Bintapu 25.05.1980 (LE)	Caucasus, Russia	KP862580, KP862568, KP862556
***Sect*. *Trichomeae* M.L. Zhang** ^b^			
*C*. *sinica* Rehd. 1	J.F. Huang 2010020 (XIJI)	Jiaocheng, Shanxi, China	KP862572, KP862560, KP862548
*C*. *sinica* Rehd. 2	J.F. Huang 2010022 (XIJI)	Jiaocheng, Shanxi, China	KP862573, KP862561, KP862549
**Outgroups**			
***Caragana***			
**Sect. *Caragana***			
**Ser. *Caragana***			
*Car*. *arborescens* Lam.	M.L. Zhang 00–201 (PE)	Altai, Xinjiang, China	FJ537262, FJ537211, FJ537164
*Car*. *boisii* C. K. Schneid.	M.L. Zhang & Y. Kang 00–121 (PE)	Lixian, Sichuan, China	FJ537259, FJ537208, FJ537161
*Car*. *turkestanica* Kom.	M.L. Zhang 00-101(PE)	Cultivated, Bergius Botanical Garden, Stockholm, Sweden	FJ537256, FJ537206, FJ537158
**Ser. *Microphyllae* (Kom.) Pojark.**			
*Car*. *bungei* Ledeb.	M.L. Zhang et al. 99–225 (PE)	Bajanchongor, Mongolia	FJ537267, FJ537216, FJ537169
*Car*. *microphylla* Lam.	M.L. Zhang et al. 99–214 (PE)	Lhongcheng, Mongolia	FJ537264, FJ537213, FJ537166
**Sect. *Bracteolatae* (Kom.) M. L. Zhang**			
**Ser. *Bracteolatae* Kom.**			
*Car*. *bicolor* Kom.	M.L. Zhang & Y. Kang Y 99–178 (PE)	Markang, Sichuan, China	FJ537246, FJ537197, FJ537147
*Car*. *brevispina* Benth.	M.L. Zhang 281-05-8414/101 (PE)	Cultivated, Berlin Botanical Garden, Germany (originally from Kashmir)	FJ537248, FJ537200, FJ537150
*Car*. *sukiensis* C. K. Schneid.	S.G. Miehe & K. Kock s.n. (NHM)	Donkardzong, Nepal	FJ537247, FJ537199, FJ537149
**Sect. *Jubatae* (Kom.) Y. Z. Zhao**			
**Ser. *Jubatae* Kom.**			
*Car*. *jubata* (Pall.) Poir.	M.L. Zhang 00279 (PE)	Zhaosu (Tianshan), Xinjiang, China	FJ537242, FJ537194, FJ537143
*Car*. *pleiophylla* (Regel) Pojark.	M.L. Zhang 10–146 (PE)	Tekes, Xinjiang, China	FJ537253, FJ537203, FJ537155
**Ser. *Leucospinae* Y. Z. Zhao**			
*Car*. *tibetica* (Maxim. ex C. K. Schneid.) Kom.	M.L. Zhang 00–89 (PE)	Uhai, Nei Mongol, China	FJ537244, FJ537195, FJ537145
**Sect. *Frutescentes* (Kom.) Sanchir**			
**Ser. *Frutescentes* Kom.**			
*Car*. *kirghisorum* Pojark.	C.Y. Chang et al. 2004219 (WUG)	Khorgos, Xinjiang, China	FJ537820, FJ537229, FJ537181
*Car*. *opulens* Kom.	M.L. Zhang & Y. Kang 99–123 (PE)	Daofu, Sichuan, China	FJ537282, FJ537231, FJ537183
**Ser. *Chamlagu* Pojark.**			
*Car*. *rosea* Turcz. ex Maxim.	M.L. Zhang 99–45 (PE)	Beihuashan, Beijing, China	FJ537272, FJ537221, FJ537174
*Car*. *sinica* (Buc’hoz) Rehder	M.L. Zhang 99–49 (PE)	Xiangshan, Beijing, China	FJ537284, FJ537233, FJ537185
**Ser. *Pygmaeae* Kom.**			
*Car*. *brevifolia* Kom.	Q.L. Ho et al. 2498 (NHM)	Yushu, Qinghai, China	FJ537268, FJ537217, FJ537170
*Car*. *stenophylla* Pojark.	M.L. Zhang 00–78 (PE)	Hangjinqi, Nei Mongol, China	FJ537274, FJ537223, FJ537176
*Car*. *versicolor* Benth.	S. Miehe 99-62-06 (NHM)	Upper Dolpo, Nepal	FJ537271, FJ537220, FJ537173
**Sect. *Spinosae* (Kom.) Y. Z. Zhao**			
**Ser. *Spinosae* Kom.**			
*Car*. *hololeuca* Bunge ex Kom.	M.L. Zhang 00–153 (PE)	Cultivated, Turfan Botanical Garden, Xinjiang, China	FJ537240, FJ537192, FJ537141
*Car*. *spinosa* (L.) Hornem.	C.Y. Chang et al. 2004503 (WUG)	Qinghe, Xinjiang, China	FJ537241, FJ537193, FJ537142
**Ser. *Acanthophyllae* Pojark.**			
*Car*. *acanthophylla* Kom.	M.L. Zhang 00–154 (PE)	Cultivated, Turfan Botanical Garden, Xinjiang, China	FJ537238, FJ537191, FJ537139
*Halimodendron halodendron* (Pall.) Voss	M.L. Zhang 00–279 (PE)	Cultivated, Urumqi Botanical Garden, Xinjiang, China	FJ537289, FJ537237, FJ537190
*Hedysarum alpinum* L.	M. Riewe 182 (CAS)	Northwest Territories, Canada	FJ537287, FJ537235, FJ537188
*Astragalus coluteocarpus* Boiss.	Qinghai-Xizang Expedition Team 76–8083 (PE)	Zada, Ali, Xizang, China	FJ537286,————, FJ537187

Classification system of *Caragana* follows Zhang (1997) (See also Zhang et al. 2009, Table 1). Classification system of *Calophaca* follows Gorbanova (1987) concerning two sections, and a new section, Sect. *Trichomeae* M.L. Zhang is yielded from this paper.

A previous phylogenetic study of the nuclear ITS region and two chloroplast regions (*trnS-trnG* and *rbcL*) indicated *Calophaca* to be nested within *Caragana* [27], but only one species was sampled from *Calophaca*. Here, sampling all of the species of *Calophaca*, we conduct a phylogenetic analysis to assess its systematic position, monophyly, and inter-species relationships, and its putative geographical origin and dispersion across Eurasia.

## Materials and Methods

### Taxon sampling

Fourteen individuals representing all eight species currently recognized in *Calophaca* were sampled for this study [14,17,25], shown in Table 1 and deposited in the herbaria. According to the previous phylogenetic studies [27–30], *Calophaca* and *Halimodendron* nest within *Caragana*. Thus the genera *Halimodendron*, *Caragana*, *Hedysarum*, and *Astragalus* were included as outgroups in this study (Table 1), with *Astragalus* serving to root the trees. To assess the monophyly of *Calophaca*, 21 species of the genus *Caragana* were also sampled, giving a total of 38 samples that were included in the study. All species materials vouchers in Table 1 are deposited in the public herbaria PE (Institute of Botany, Chinese Academy of Sciences, Beijing, China), XIJI (Xinjiang Institute of Ecology and Geography, Chinese Academy of Sciences, Urumqi, China), WUG (Northwest Institute of Botany, Yangling, Shaanxi, China), LE (Komarov Botanical Institute, Russian Academy of Sciences, St. Petersburg, Russia), MW (Moscow University, Moscow, Russia) and MAH (Main Botanical Garden, Russian Academy of Sciences, Moscow, Russia).

We state that all use of species materials were permitted by the authority of these herbaria. Our study did not concern Human Subject Research or Animal Research. We can declare that the leaf materials did not come from conservation parks, and none of the samples involved endangered or protected species.

### DNA sequencing

Total genomic DNA was extracted using the CTAB method [31]. For primers of the ITS region, *trnS*-*trnG* spacer and *rbcL*, see Zhang et al. [27]. The polymerase chain reaction (PCR) was used for amplification of double stranded DNA. The 25 μl reaction system contained 0.25 μl of Ex *Taq*, 2.5 μl of 10× Ex Taq buffer (Mg^2+^ concentration of 25 mM), 2.0 μl of dNTP mix (2.5 mM concentration for each dNTP), 1 μl of the forward and reverse primers at 5 umol/μl, and 0.5 μl of template DNA. The protocol for amplification consisted of an initial hot start at 95°C for 2 min, followed by 30 cycles of denaturing at 94°C for 30 s, annealing at 52°C for 30 s, extension at 72°C for 90 s, and a final extension at 72°C for 10 min. PCR products were purified using the PEG precipitation procedure [32] and sequenced using an ABI Prism 3770 Genetic Analyzer (Shanghai Shenggong Biological Engineering Technology & Service, Shanghai, China).

Sequences were aligned with CLUSTAL X [33] and then adjusted manually. All gaps were treated as missing data cells. Finally, the combined 3-genic region data set comprised 3417 aligned nucleotide characters.

### Phylogenetic analysis and divergence time estimate

The incongruence length difference (ILD) test [34] was carried out in PAUP* [35] to assess potential conflicts between data set partitions. This was implemented with 100 partition-homogeneity test replicates, a heuristic search option with simple addition of taxa, TBR branch swapping, and MaxTrees set to 1000. Phylogenetic analyses were performed using maximum likelihood (ML) and Bayesian inference. ML analysis was implemented with PAUP; clade support was estimated with 1,000 heuristic bootstrap replicates (100 random addition cycles per replicate, with tree bisection-reconnection and branch-swapping) [36,37]. For ML analysis, Modeltest 3.06 [38] was used to estimate the appropriate model of DNA substitution for sequence data. The models selected by the Akaike information criterion (AIC) were TIM+I+G. The related parameters of Modeltest were used for the ML analysis.

Bayesian phylogenetic analysis and divergence time estimates were implemented in BEAST 1.5.4 [39,40]. We used the uncorrelated lognormal relaxed clock model with a Yule process for the speciation model, and GTR+I+G for the substitution model (estimated for the data set). The age of tree prior (normal distribution Mean = 28, Stdev = 1) for Hedysareae and Astragaleae, i.e., the ancestral node of *Astragalus* and *Caragana* was defined as the root at 28 Ma following Lavin et al. [41], Wojciechowski [30] and Zhang and Fritsch [42]. A Markov chain Monte Carlo was run for 50 million generations and sampled every 1,000 generations. Two independent runs were performed to confirm convergence of the analysis. The stationarity of each run was examined using the effective sampling size of each parameter (>200). The last 40 million generations were used to construct the maximum clade credibility tree and the associated 95% highest posterior density distributions around the estimated node ages using program TreeAnnotator 1.5.4, and visualized using FigTree 1.3.1.

### Biogeographical analysis

We designated five biogeographical areas based on the distributions of the *Calophaca* species, especially its disjunctions, see Fig 1. Most of the species occurring in these areas are endemic, for example, *C*. *grandiflora* and *C*. *sericea* are endemic to the Pamir mountains, *C*. *wolgarica* to the Caucasus, and *C*. *sinica* to East Asia. The areas chosen are natural regions, and often independent biodiversity hotspots [19], for example, the Pamir and Tianshan mountains and the Caucasus. These areas also have distinct vegetation and floras; especially different are those of the Pamir, western Tianshan, and Tarbagatai mountains, see Fig 1. The five designated areas are thus: **A**: the Pamirs, Tadjikistan; **B**: the western Tianshan Mountains, including portions of Kyrgistan and Kazakhstan; **C**: the Tarbagatai, including portions of northwestern China and Kazakhstan; **D**: East Asia, and montane portions of Shanxi Province, China; **E**: the Caucasus, and plains along the Volga and Don Rivers.

To infer vicariance and dispersal events, a Bayesian parsimony-based method (S-DIVA) and BBM (Bayesian Binary Method) were employed [43,44]. DIVA is an event-based method that optimizes ancestral distributions by assuming a vicariance explanation, while incorporating the potential contributions of dispersal and extinction [45]. Nylander et al. [46] proposed a modified approach to DIVA named Bayes-DIVA that integrates biogeographical reconstructions of DIVA over the posterior distribution of a Bayesian MCMC sample of tree topologies. Bayes-DIVA is also referred to as S-DIVA [43]. BBM infers ancestral areas using a full hierarchical Bayesian approach; it hypothesizes a special “null distribution” which means that an ancestral range contains none of the unit areas [44]. S-DIVA and BBM can be performed in RASP (Reconstruct Ancestral State in Phylogenies) 2.0 beta. http://mnh.scu.edu.cn/soft/blog/RASP.

**Fig 2:**
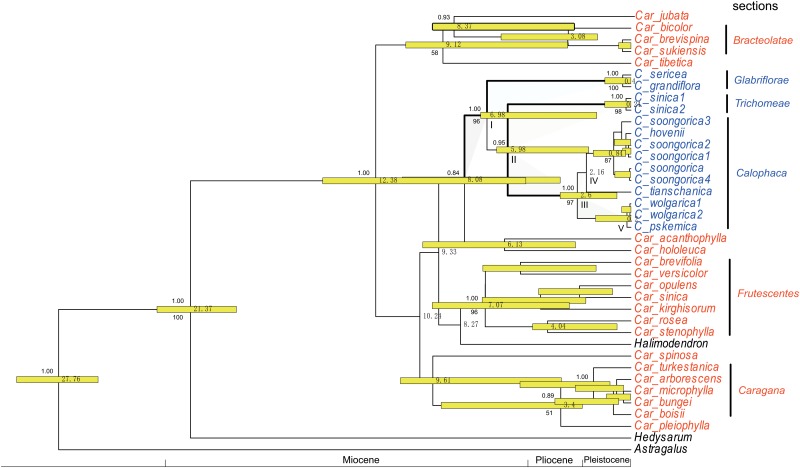
Chronogram of *Calophaca* and outgroups *Caragana*, *Halimodendron*, *Hedysarum*, and *Astragalus*, with maximum clade credibility performed by BEAST. Dates of divergence are shown to the right of nodes, and posterior probability values are shown to the left of nodes.

The BEAST molecular dating tree (Fig 2) was treated as a fully resolved phylogram for use as the basis for S-DIVA and BBM, with 1000 post-burnin trees derived from the BEAST analysis used for ancestral area reconstruction in the program RASP. Multiple samples of the same taxon in a clade are combined into one branch, or many identical terminal areas in a clade are regarded as one; consequently, the tree is reduced to maximum simplicity. RASP was performed with various constraints of maximum areas 2 at each node, to infer possible ancestral areas and potential vicariance and dispersal events [47–49].

**Fig 3:**
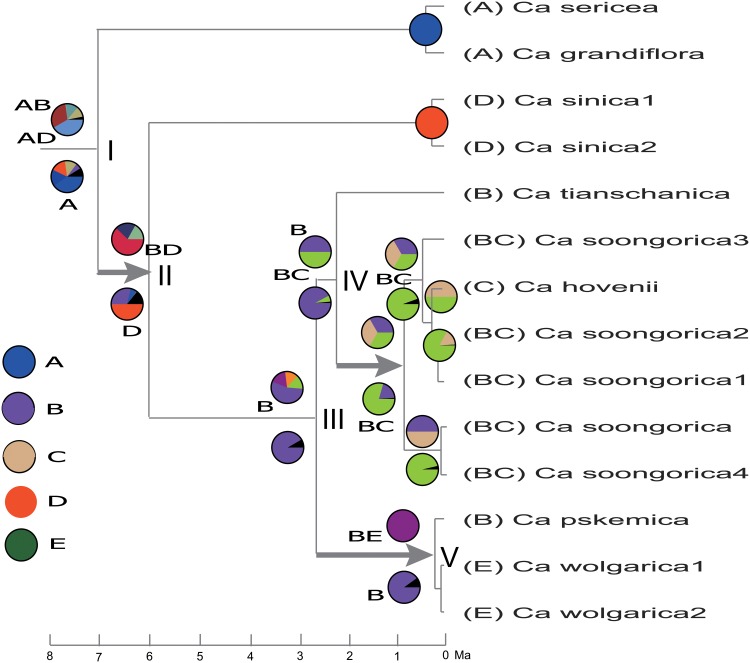
Ancestral area optimizations performed with S-DIVA and BBM. Pie chartsat nodes are conducted from S-DIVA (on the branch above) and BBM (on the branch below). The three thicked arrowheaded lines at branches show four dispersals. Area labels, as stated in the text: **A**: Pamir Mountains, Tadjikistan; **B**: western Tianshan Mountains, plus parts of Kyrgistan and Kazakhstan; **C**: Tarbagatai Mountains, including adjacent regions of China and Kazakhstan; **D**: East Asia, mountains of Shanxi Province, China; **E**: Caucasus, and plains along the Volga River and Don River.

The outgroups used in the phylogeny were also used in the biogeographical analyses throughout data processing, but are not shown in the resulting Fig 3.

## Results

### Phylogenetic analysis and divergence time estimate

The data partitions of the three genes were not significantly incongruent on the basis of the ILD tests (P = 0.133). ML analysis yielded three optimal trees. Topologies of the three were on the whole equivalent to the Bayesian BEAST tree. Since the BEAST tree also has a dating result, it is used to illustrate phylogenetic relationships and the dating results (see Fig 2).

Our phylogenetic analyses show that *Calophaca* is monophyletic, with high bootstrap (bt) support and posterior probability (pp) (bt = 96%, pp = 1.00) (node I), and is nested within *Caragana* (Fig 2). The phylogenetic analysis yielded a topology largely in accordance with the current infrageneric classification of *Calophaca*. The two species of *C*. sect. *Glabriflorae* formed a clade (bt = 100%, pp = 1.00), which is sister to the other taxa, and the two samples of *C*. *sinica* also formed a clade (bt = 98%, pp = 1.00). Samples of *C*. *soongorica*, *C*. *hovenii*, *C*. *pskemica*, *C*. *wolgarica*, and *C*. *tianshanica* comprised a clade (bt = 97%, pp = 1.00) (node III), equivalent to section *Calophaca*. These three clades within *Calophaca* had high bootstrap and posterior probability values (Fig 2).

The estimated divergence (stem) age of *Calophaca* was ca. 8.0 (95% HPD: 3.44–11.09) Ma, the crown age between the Pamirian sect. *Glabriflorae* and the other two sections was ca. 7.0 (95% HPD: 1.66–7.29, crown age) Ma (node I), and the age between the East Asian sect. *Trichomeae* and the Tianshan Mountains sect. *Calophaca* was ca. 6.0 Ma (Fig 2, node II, crown age). The diversification time range within sect. *Calophaca* was from Pliocene to Pleistocene, i.e., 2.6–0.2 Ma (Fig 2, node III). The diversification time of the two species of sect. *Glabriflorae* was ca. 0.4 Ma. *Calophaca wolgarica*, distributed from the Caucasus to Russian Europe, was formed more recently at ca. 0.2 Ma.

### Ancestral area reconstructions

Within *Calophaca* sect. *Calophaca*, the ancestral area (node III, Fig 3) estimated from S-DIVA (B and BC at equal probability) and BBM (B), thus we chose it as B (western Tianshan Mountains). The remaining area C (Tarbagatai Mountains) within this section (Fig 3), is shown as a dispersal many times. The most incongruent and uncertain node of the ancestral distribution estimates is at the root of *Calophaca* (node I). Considering S-DIVA, showing the likely ancestral area as AD (union of Pamir Mountains and East Asia) and AB (union of the Pamir and western Tianshan mountains), A is the intersection, occurring in both ancestral areas. Concerning the value of AD at node I, and the reason for the selection of A (ignoring D), was that we mainly thought A, with *C*. *sericea* and *C*. *grandiflora* at the phylogenetic tree base (stem age ca. 6.98 Ma, see Fig 2), to be more “old” than D of *C*. *sinica* (stem age ca. 5.98 Ma). This indicates that A (Pamir) should be the ancestral area instead of D (East Asia). In addition, ignoring AD as an ancestral area would mean ignoring an impossibly large ancestral area union, covering two separate portions, the Pamir and East Asia (North China, Shanxi Province), and consequently the first speciation of *Calophaca* would have had to be speculated as a vicariance between Pamir and East Asia, in contrast with the unique place of origin of the Pamirs. Therefore, we felt we should choose the single area A as ancestral. In addition, BBM, with a most likely estimated area of A, confirmed and justified the S-DIVA result. Therefore, it appeares preferable to choose A as the ancestral area of *Calophaca*.

After deciding the ancestral areas at the nodes (Fig 3), several dispersals can be recognized, see Figs 3 and 1. First, two dispersals are inferred from area A (Pamir) (node I) to B (western Tianshan) and D (East Asia). A dispersal is shown from node III to node V, and finally to the Caucasus. Another dispersal is from B (node IV) to C (Tarbagatai).

## Discussion

### Classification of *Calophaca*

The monophyly of *Calophaca* was verified with high bootstrap (bt) support and posterior probability (pp) (bt = 96%, pp = 1.00, node I, Fig 2). The present phylogenetic tree (Fig 2) indicates three clades with high support within *Calophaca*, one consisting of *C*. *grandiflora* and *C*. *sericea*, a second containing *C*. *sinica*, and a third containing *C*. *soongorica*, *C*. *tianschanica*, *C*. *hovenii*, *C*. *wolgorica*, and *C*. *pskemica*. This is well consistent with previous morphological classification except for *C*. *sinica*. According to Gorbunova’s [15] classification system for the genus, *C*. *grandiflora* and *C*. *sericea* are included in sect. *Glabriflora*, while *C*. *soongorica* and *C*. *wolgorica*, etc. belong to sect. *Calophaca*. *Calophaca sinica* has a combination of distinctive characters, with dense glandular trichomes on the standard, peduncle, calyx, ovary, style, and a legume different from other species within the genus, and has an endemic distribution in East Asia. Therefore, we can use it to establish a new section Trichomeae, to contrast with the two sections of Gorbanova [15], see S1 File. This effectively completes the classification for the genus, with a total of three sections.

The five accessions of *Calophaca soongorica* formed a clade with the inclusion of *C*. *hovenii*. *Calophaca hovenii* Schrenk, *C*. *tianshanica* (B. Fedtsch.) Borissova, and *C*. *soongorica* (*C*. *soongorica* 1 and 2 = *C*. *chinensis* Borissova, see Table 1) were included within *C*. *soongorica* as varieties [14,15,18]. Clearly, *C*. *hovenii* and the others should be placed within *C*. *soongorica*.

In the traditional taxonomy, *Calophaca*, *Halimodendron*, and *Caragana* are regarded as related and distinctive genera [20,21]. Previously the three were included in subtribe Astragaleae of tribe Galegeae [20], and later changed to tribe Hedysareae [21]. Diagnostic characters of *Calophaca* and *Caragana* are that while both are shrubs with pinnate leaflets, *Calophaca* is imparipinnate but *Caragana* is paripinnate. Contrasting with the flat pods of *Calophaca* and *Caragana*, *Halimodendron* has an inflated pod. *Calophaca*, particularly, has a distribution of montane grassland, shrubland, and dry forest.

Since *Calophaca* and *Halimodendron* are nested within *Caragana* (Fig 2), it results in *Caragana* being paraphyletic. In terms of the taxonomic identities of *Calophaca*, *Halimodendron*, and *Caragana*, *Caragana* should be subdivided into segregate taxa. Currently it has been recognized as six phylogenetic clades (Fig 2), which include the three sections labeled, as well as clades representative by *Car*. *jubata*, *Car*. *acanthophylla*, and *Car*. *spinosa*. However, in view of the limitation of sampled taxa in our study, further research should be conducted examining sufficient taxa and more genes. Essentially, the present paper focuses on *Calophaca*.

### Origin and diversification

Our results yielded a divergence time (stem age, or origin age) of ca. 8.0 Ma for *Calophaca* (Fig 2), and a place of origin in the Pamir Mountains (Fig 3), which implies that a global cooling and drying process at 8–7 Ma in the late Miocene [10,13], most likely drove generic origin. It is also possible more local events played a part in the origin, especially major tectonism in the Tianshan range at about 6.5 Ma [50,51]; the Pamir mass had experienced severe deformation and uplift from the India-Asia impact at a previous time [52]. Progressive aridification in connection with global cooling appears to have played an important role in the later Miocene [13]. The Pamir-western Tianshan mountains experienced progressively more arid climates. This climate transition possibly led to range restriction and isolation of organisms, and *Calophaca* may be one of the many lineages that then evolved and diversified in the Pamirs.

Compared with the diversification time of *Caragana* [42], the crown age (diversification age) of *Calophaca* at ca. 6.98 Ma coincided with the diversification times of the three sections within *Caragana*, whereas diversification times of the three *Calophaca* sections are younger at 2.6, 0.4 and 0.24 Ma (Fig 2). Most Central Asian *Calophaca* species, such as *C*. *grandiflora*, *C*. *sericea*, *C*. *soongorica*, and *C*. *wolgarica*, have young crown ages i.e., Pliocene to Pleistocene 0.84–0.2 Ma (Fig 2). These show that the genus is a relatively young taxon among lineages of temperate legume shrubs and the Central Asian flora.

In general, the Central Asian flora is speculated to have formed from Tertiary Tethys relicts [12]. *Hippophae rhamnoides* (Elaeagnaceae) distributed in Eurasia, was similarly shown to have originated from the QTP and migrated to Central Asia and Asia Minor ⁄ Europe [6]. *Artemisia* has been shown to have originated from the arid-semiarid middle latitudes of Asia in the late Eocene, and then spread westward and eastward in the Oligocene as the result of early QTP uplift and accompanying aridification [9]. *Calophaca* presents another pattern of Asian origin and dispersal that is somewhat different from that of *Hippophae rhamnoides* and *Artemisia*.

Parts of the QTP may have experienced rapid uplift contributing to global cooling during past 8–7 Ma [13,53–60]. Responding to this QTP uplift event and global cooling and drying, many biological cases mainly occurring in situ in the QTP have been contributed, for example, the *Ligularia Cremanthodium—Parasenecio* complex (Asteraceae) [61], *Rheum* (Polygonaceae) [62], *Androsace* (Primulaceae) [3] and *Saussurea* (Asteraceae) [63], and the glyptosternoid catfishes [64]. Even though absent in the QTP, *Calophaca* has a diversification crown age of ca. 6.98 Ma and a distribution located to the north of the QTP, and it can be supposed that the origin and evolution of *Calophaca* were driven by the same paleogeographic and paleoclimatic conditions.

### Dispersal and disjunction

According to the biogeographical analysis (Fig 3), we can hypothesize that dispersal may be a dominant speciation mode in *Calophaca*. There are four remarkable dispersals from the late Miocene to Pleistocene in this genus, i.e., from the Pamir to the western Tianshan mountains, and to East Asia at ca. 7.0 Ma, northward to the Tarbagatai Mountains at ca. 2.2 Ma, and from the western Tianshan Mountains westward to the Caucasus—Russian Europe at 2.6 Ma (Fig 1). The great arid and semiarid belt, comprising the Tarim Basin, Junggar Basin, Hexi Corridor, and Loess Plateau in arid northwestern China forms a barrier between the Pamir-western Tianshan mountains and East Asia. Similarly, the Turan lowland and desert separates the Pamir—western Tianshan mountains and the Caucasus. The presence of these arid belts most likely resulted from the intensified Asian Interior aridification which is associated with global cooling and drying since the Middle Miocene optimum 8–7 Ma [13], and consequently to have fragmented the ranges of the *Calophaca* species and also eliminated the traces of their dispersals.

### Montane distribution and refugia

Extant *Calophaca* species of the Pamir and western Tianshan mountains, such as *C*. *grandiflora*, *C*. *sericea*, *C*. *tianshanica*, *C*. *pskemica*, and *C*. *soongorica*, often occur in middle or low montane belts at altitudes of 800–2800 m, sometimes in meadows or steppe, with most individuals occurring in juniper forests or shrublands [14,17]. *Calophaca sinica*, in the northern China mountains, likewise grows in forest or shrubland [65]. *Calophaca wolgarica*, a shrub endemic to the Caucasus and eastern Europe, occurs on steppe, plains, and limestone hills [14,17]. In conclusion, the predominant habitat of the genus is the lower montane, in the Pamir—western Tianshan mountains, the Caucasus, and the northern China mountains.

These montane distribution centers of *Calophaca*, occupy several global biodiversity hotspots [19], especially the Pamir—western Tianshan mountains and the Caucasus. These areas are also regarded as plant refugia of the Quaternary, since the mountains have maintained a somewhat moist habitat, and thus have an advantage in protection of species from destruction caused by enhanced cooling and drying in the Quaternary [66–73]. The high level of genetic and taxonomic diversity of this area is indicative of its ecological stability [67,69]. Therefore, *Calophaca* is in fact a representative of the global biodiversity hotspots and refugia.

## Supporting Information

S1 File New section within *Calophaca*. (PDF)
