# Supplementary material for: Spatiotemporal Evolution of Calophaca (Fabaceae) Reveals Multiple Dispersals in Central Asian Mountains
Source: PLoS One. 2015 Apr 7;10(4):e0123228. doi: 10.1371/journal.pone.0123228 (PMC4388477; doi:10.1371/journal.pone.0123228)
Supplement: S1 File — (PDF) [file pone.0123228.s001.pdf]

S1 New section within *Calophaca*

Section *Trichomeae* M.L. Zhang stat. nov.

With dense glandular trichomes on the standard, peduncle, calyx, ovary, style, and legume. Stipules longer, seeds ellipitical.

Type: *C. sinica* Rehd.

This section has only one species, endemic to the Taihangshan and Lüliangshan Mountains of Shanxi Province and the southern Yinshan Mountains of Inner Mongolia, occurring in forest and shrubland, altitude 900-1400 m. In Inner Mongolia, the habitats have been destroyed on a tremendous scale, thus, this species is regarded as rare and endangered and to be conserved in China.
